# P02.156. Effects of a gentle yoga program on Restless Legs Syndrome (RLS) symptoms and related outcomes in women with RLS: a pilot study

**DOI:** 10.1186/1472-6882-12-S1-P212

**Published:** 2012-06-12

**Authors:** K Innes, T Selfe, K Leeming, P Agarwal

**Affiliations:** 1 West Virginia University, Morgantown, USA

## Purpose

RLS is a common sleep disorder that negatively affects health, well-being, and quality of life. Although standard drug treatments for RLS can carry serious side effects, promising nonpharmacologic therapies, while widely recommended, remain little investigated. In this pilot pre-post trial, we assessed the effects of a gentle 8-week yoga program on RLS symptoms and related outcomes in women with RLS.

## Methods

Participants were 13 women with moderate-severe RLS (International RLS Study Group criteria) who had not been diagnosed with diabetic neuropathy or other serious conditions; did not suffer from another sleep disorder; and were not taking RLS medication. All reported RLS symptoms at least 2 days/week. The intervention was an 8-week Iyengar yoga program. All participants attended two 90-minute classes per week and were asked to perform 30 minutes of home practice on non-class days. Primary outcomes assessed pre- and post-treatment were RLS symptoms and symptom severity (IRLS scale) and sleep quality (MOS-Sleep Scale). Secondary outcomes included mood [Profile of Mood States (POMS)] and perceived stress [Perceived Stress Scale (PSS)].

## Results

Ten women (mean age 49.5±3.9, range 32-66 years; mean BMI=29.6±2.3) completed the study. Compliance was excellent overall; participants attended an average of 13.4±0.3 classes and completed an average of 4.1±0.1 homework sessions/week. Participants demonstrated a 49% decline in RLS symptoms overall (p=0.01), a 62% decrease in symptom severity (p=0.0006), as well as significant improvement in sleep, both overall (p<0.0005), and in 3 of 4 primary domains [sleep disturbance, sleep adequacy, and somnolence (p<0.003)]. Participants also showed declines in perceived stress (p<0.02) and multiple domains of mood.

## Conclusion

These preliminary findings suggest that yoga may offer a safe, acceptable, and effective therapy for attenuating RLS symptoms, and improving sleep and mood in women with RLS. Larger controlled trials are needed to confirm and further investigate the potential benefits of yoga for RLS management.

